# Maize defense elicitor, 12-oxo-phytodienoic acid, prolongs aphid salivation

**DOI:** 10.1080/19420889.2020.1763562

**Published:** 2020-05-13

**Authors:** Sajjan Grover, Suresh Varsani, Michael V. Kolomiets, Joe Louis

**Affiliations:** a Department of Entomology, University of Nebraska-Lincoln, Lincoln, NE, USA; b Department of Plant Pathology and Microbiology, Texas A & M University, College Station, TX, USA; c Department of Biochemistry, University of Nebraska-Lincoln, Lincoln, NE, USA

**Keywords:** Corn leaf aphid, maize, 12-OPDA, callose, EPG

## Abstract

12-﻿Oxo-phytodienoic acid (OPDA), an intermediate in the jasmonic acid (JA) biosynthesis pathway, regulates diverse signaling functions in plants, including enhanced resistance to insect pests. We previously demonstrated that OPDA promoted enhanced callose accumulation and heightened resistance to corn leaf aphid (CLA; *Rhopalosiphum maidis*), a phloem sap-sucking insect pest of maize (*Zea mays*). In this study, we used the electrical penetration graph (EPG) technique to monitor and quantify the different CLA feeding patterns on the maize JA-deficient *12-oxo-phytodienoic acid reductase* (*opr7opr8*) plants. CLA feeding behavior was unaffected on B73, *opr7opr8* control plants (- OPDA), and *opr7opr8* plants that were pretreated with OPDA (+ OPDA). However, exogenous application of OPDA on *opr7opr8* plants prolonged aphid salivation, a hallmark of aphids’ ability to suppress the plant defense responses. Collectively, our results indicate that CLA utilizes its salivary secretions to suppress or unplug the OPDA-mediated sieve element occlusions in maize.

The corn leaf aphid (CLA; *Rhopalosiphum maidis*), a piercing-sucking insect pest, is one of the most damaging pests of many cereal crops, including maize (*Zea mays*) [1–6]. Unlike chewing herbivores, CLA feeds by inserting their slender stylets into phloem sieve elements to consume the nutrients required for their growth and development. CLA feeding also transmits various plant viral diseases [2,7,8]. In addition, the aphid honeydew, the digestive waste produced by aphids, which are deposited on the leaves promotes sooty mold growth, thereby reducing the photosynthetic efficiency of plants [9].

We have previously shown that 12-oxo-phytodienoic acid (OPDA), an intermediate in the jasmonic acid (JA) biosynthesis pathway, promotes heightened maize resistance against aphids [6]. In addition, exogenous application of OPDA enhanced callose accumulation, one of the defense mechanisms utilized by plants against insect attack, and also enhanced the expression of ethylene biosynthesis and receptor genes that act as an important modulator in regulating *maize insect resistance1* (*mir1*)-dependent maize defense to CLA [5,6]. However, artificial diet aphid bioassays confirmed that OPDA does not have a direct negative impact on CLA population, rather the OPDA-induced activation of downstream defenses contributed to enhanced maize resistance to CLA [6].

## Exogenous application of OPDA does not affect the feeding of CLA on maize plants

**Figure 1.**
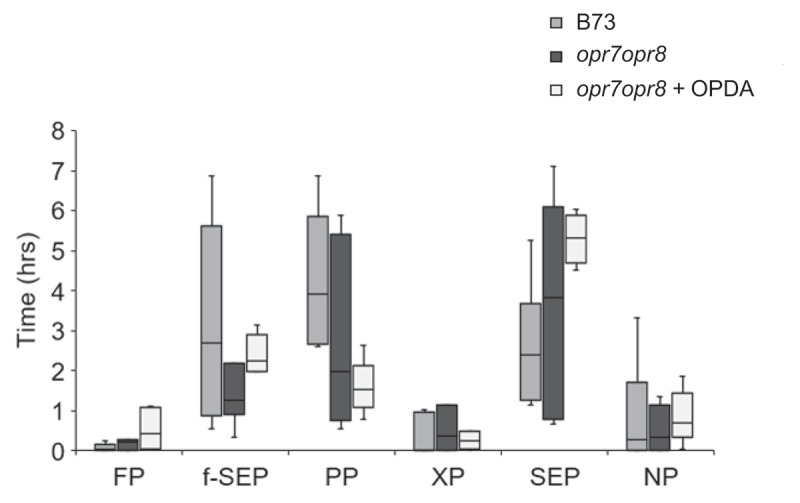
Electrical penetration graph (EPG) comparison of time spent by CLA in various feeding activities on maize B73, *opr7opr8*, and *opr7opr8* plants pretreated with OPDA in 8 h of recording time. FP, time taken to the first probe; f-SEP, time taken to reach first sieve element phase; PP, time spent in pathway phase; XP, total time spent in the xylem phase; SEP, total time spent in the sieve element phase or phloem phase; NP, total time spent in nonprobing phase during the 8 h recording time. Boxplots represent median and range for each treatment (n = 5–7). EPG was analyzed by the non‐parametric Kruskal–Wallis test. Statistically significant differences were not observed among any of the aphid feeding parameters on B73, *opr7opr8*, and *opr7opr8* plants pretreated with OPDA.

In maize, two *12-Oxo-Phytodienoic acid Reductase* (*OPR7* and *OPR8*) genes are involved in the conversion of OPDA to JA [10]. Basal and wound-induced OPDA levels in *opr7 opr8* double mutants were reduced as compared to wild-type B73 plants, whereas JA induction was undetectable in *opr7opr8* plants [11]. Previously, we showed that there were comparable CLA numbers on B73 and *opr7opr8* plants, however, exogenous application of OPDA showed significantly lesser aphid numbers on *opr7opr8* plants [6]. Similarly, exogenous application of OPDA and feeding by CLA on *opr7opr8* plants increased the callose accumulation compared to *opr7opr8* control plants and wild-type plants [6]. These findings suggested that the OPDA-mediated resistance to CLA in maize can occur independently of the JA pathway and signaling mechanisms. Strong antibiosis, which curtails insect fecundity and population growth, can also influence insect’s feeding behavior [9]. To determine if exogenous OPDA application can affect CLA feeding behavior, we utilized the electrical penetration graph (EPG) technique [6,12–14] to monitor and quantify the different CLA feeding activities on *opr7opr8* plants. Using EPG, the various parameters measured included the time taken to first probe (FP), time taken to reach first sieve element phase (f-SEP), time spent in the pathway phase that represent both the inter- and/or intracellular aphid stylet routes during feeding (PP), total time spent in the SEP, total time spent in the xylem phase (XP), and total time spent in nonprobing phase (NP). As shown in Figure 1, there were no significant differences in any of these parameters measured for the CLA feeding behavior on the wild-type (B73), *opr7opr8* control plants (- OPDA) and OPDA pretreated *opr7opr8* (+ OPDA) plants. The EPG result suggests that OPDA pretreatment does not have an effect on aphid feeding behavior.

## OPDA pretreatment extends aphid salivation on maize plants

**Figure 2.**
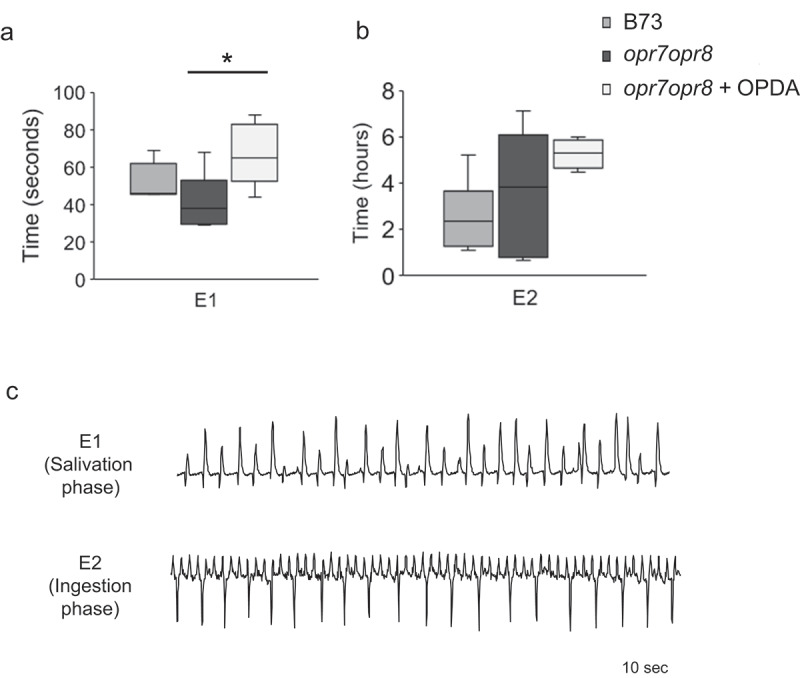
Electrical penetration graph (EPG) comparison of time spent by CLA in the E1 (salivation) (a) and E2 (ingestion) (b) phases during the sieve element phase (SEP) on maize B73, *opr7opr8*, and *opr7opr8* plants pretreated with OPDA in 8 h of recording time. Boxplots represent median and range for each treatment (n = 5–7). Asterisks indicate significant difference (*P* < 0.05; Kruskal–Wallis test) among individual CLA feeding parameters on different maize plants. (c) Representative EPG waveform patterns of E1 and E2 during the CLA feeding on maize plants for 10 seconds.

The SEP consists of E1 (salivation) and E2 (sap ingestion) phases [15]. E1 phase, the initial phase in the SEP, represents aphid salivation and in general, could remain approximately for one minute. E2 waveform represents subsequent ingestion of phloem sap with continuous salivation and it could range from several minutes to hours [15]. Aphids secrete watery saliva during E1 SEP, which contains salivary effectors that alter host physiology for their own benefit and to assist continued feeding from the sieve elements, before start ingesting phloem sap (E2) [9,15–17]. Our results indicate that CLA spent a significantly longer time in the E1 phase of OPDA pretreated *opr7opr8* (+ OPDA) plants compared to the wild-type (B73) and *opr7opr8* control plants (- OPDA) (Figure 2(a)). In contrast, there was no significant difference in the E2 phase of CLA feeding on the wild-type (B73), *opr7opr8* control plants (- OPDA) and OPDA pretreated *opr7opr8* (+ OPDA) plants (Figure 2(b)). Figure 2(c) shows the representative E1 and E2 waveform patterns produced by CLA feeding on maize plants.

Upon aphid feeding, as a counter-defense mechanism, plants induce the phloem wound responses, such as aggregation of phloem proteins and callose deposition [15,18]. It should also be noted that the wound responses in sieve elements by aphid stylets compared to severing the sieve elements by a glass needle, which mimics aphid feeding, are distinct and do not lead to the activation of similar set of phloem proteins [15]. Furthermore, studies have shown that an extended E1 phase is indicative of the ability of the aphids to suppress the wound defense responses induced by insect feeding [15,19]. It was previously shown that OPDA pretreatment enhanced callose accumulation on maize plants [6]. It is highly likely that CLA may inject more watery saliva into the sieve elements to suppress the defense responses, for example, suppression of sieve element occlusion by dissolving callose accumulation. However, it remains unclear how aphid salivation suppresses OPDA-mediated defenses. Ca^2+^ is reported to have a major role in phloem occlusion through its effect on callose deposition and coagulating phloem proteins [20–22]. Ca^2+^ binding proteins are identified in the salivary glands of aphids [23], suggesting that aphids may inject these proteins during E1 phase to suppress the wound responses. Whether similar Ca^2+^ binding proteins and/or other salivary gland proteins are required for E1 salivation in CLA are yet to be determined.

## References

[1] Bing JW, Guthrie WD. Generation mean analysis for resistance in maize to the corn leaf aphid (Homoptera: aphididae). J Econ Entomol. 1991;84:1080–1082.

[2] Carena MJ, Glogoza P. Resistance of maize to the corn leaf aphid: A review. Maydica. 2004;49:241–254.

[3] Meihls LN, Handrick V, Glauser G, et al. Natural variation in maize aphid resistance is associated with 2,4-dihydroxy-7-methoxy-1,4-benzoxazin-3-one glucoside methyltransferase activity. Plant Cell. 2013;25:2341–2355. 23898034 10.1105/tpc.113.112409 PMC3723630

[4] Pointeau S, Jaguenet E, Couty A, et al. Differential performance and behavior of the corn leaf aphid, *Rhopalosiphum maidis*, on three species of the biomass crop *Miscanthus*. Ind Crops Prod. 2014;54:135–141.

[5] Louis J, Basu S, Varsani S, et al. Ethylene contributes to *maize insect resistance1*-mediated maize defense against the phloem sap-sucking corn leaf aphid. Plant Physiol. 2015;169:313–324. 26253737 10.1104/pp.15.00958 PMC4577432

[6] Varsani S, Grover S, Zhou S, et al. 12-Oxo-phytodienoic acid acts as a regulator of maize defense against corn leaf aphid. Plant Physiol. 2019;179:01472.2018. 10.1104/pp.18.01472 PMC6446797 30643012

[7] Thongmeearkom P. Aphid transmission of maize dwarf mosaic virus strains. Phytopathology. 1976;66:332.

[8] So Y-S, Ji HC, Brewbaker JL. Resistance to corn leaf aphid (*Rhopalosiphum maidis* Fitch) in tropical corn (*Zea mays* L.). Euphytica. 2010;172:373–381.

[9] Nalam V, Louis J, Shah J. Plant defense against aphids, the pest extraordinaire. Plant Sci. 2019;279:96–107. 30709498 10.1016/j.plantsci.2018.04.027

[10] Yan Y, Christensen S, Isakeit T, et al. Disruption of *OPR7* and *OPR8* reveals the versatile functions of jasmonic acid in maize development and defense. Plant Cell. 2012;24:1420–1436. 22523204 10.1105/tpc.111.094151 PMC3398555

[11] He Y, Borrego EJ, Gorman Z, et al. Relative contribution of LOX10, green leaf volatiles and JA to wound-induced local and systemic oxylipin and hormone signature in *Zea mays* (maize). Phytochemistry. 2020;174:112334. 32172019 10.1016/j.phytochem.2020.112334

[12] Tjallingii WF Electrical recording of stylet penetration activities. In: Aphids, their biology, natural enemies and control, Vol. 2B. Amsterdam: Elsevier Science Publishers; 1988. p. 95–108.

[13] Walker GP. A beginner’s guide to electronic monitoring of homopteran probing behavior. In: Walker GP, Backus EA, editors. Principles and applications of electronic monitoring and other techniques in the study of homopteran feeding behavior. Lanham, MD: Thomas Say Publications in Entomology, Entomol. Soc. Am; 2000. p. 14–40.

[14] Louis J, Singh V, Shah J. *Arabidopsis thaliana*-Aphid Interaction. Arabidopsis Book. 2012. 10:e0159. 22666177 10.1199/tab.0159 PMC3365623

[15] Tjallingii WF. Salivary secretions by aphids interacting with proteins of phloem wound responses. J Exp Bot. 2006;57:739–745. 16467410 10.1093/jxb/erj088

[16] Hogenhout SA, Bos JIB. Effector proteins that modulate plant-insect interactions. Curr Opin Plant Biol. 2011;14:422–428. 21684190 10.1016/j.pbi.2011.05.003

[17] Kaloshian I, Walling LL. Hemipteran and dipteran pests: effectors and plant host immune regulators. J Integr Plant Biol. 2016;58:350–361. 26467026 10.1111/jipb.12438

[18] Will T, van Bel AJE. Physical and chemical interactions between aphids and plants. J Exp Bot. 2006;57:729–737. 16473888 10.1093/jxb/erj089

[19] Garzo E, Soria C, Gómez-Guillamón ML, et al. Feeding behavior of *Aphis gossypii* on resistant accessions of different melon genotypes (*Cucumis melo*). Phytoparasitica. 2002;30:129–140.

[20] Knoblauch M, van Bel AJE. Sieve tubes in action. Plant Cell. 1998;10:35–50.

[21] Knoblauch M, Peters WS, Ehlers K, et al. Reversible calcium-regulated stopcocks in legume sieve tubes. Plant Cell. 2001;13:1221–1230. 11340193 10.1105/tpc.13.5.1221 PMC135563

[22] Furch ACU, Bel AJE, van Fricker MD, et al. Sieve element Ca^2+^ channels as relay stations between remote stimuli and sieve tube occlusion in *Vicia faba*. Plant Cell. 2009;21:2118–2132. 19602624 10.1105/tpc.108.063107 PMC2729599

[23] Wang W, Dai H, Zhang Y, et al. Armet is an effector protein mediating aphid–plant interactions. Faseb J. 2015;29:2032–2045. 25678626 10.1096/fj.14-266023

